# Deep brain stimulation for dystonia: outcomes from a Brazilian cohort

**DOI:** 10.1055/s-0045-1813241

**Published:** 2026-02-04

**Authors:** Clarice Listik, Eduardo Listik, Flávia de Paiva Santos Rolim, Miriam Carvalho Soares, Renata Montes Garcia Barbosa, Bernardo Assumpção de Monaco, Marina Farah, Marcela Ferreira Cordellini, Tamine Capato, Pedro Renato de Paula Brandão, Igor Vilela Brum, Denise Maria Meneses Cury Portela, Gustavo Sousa Noleto, Ananda Falcone, Julia Carvalhinho Carlos de Souza, Sara Carvalho Barbosa Casagrande, João Carlos Papaterra Limongi, Gabriel de Castro Micheli, Lorena Souza Viana, Mariana Moscovich, Fernanda Martins Maia Carvalho, Carlos Roberto de Mello Rieder, Egberto Reis Barbosa, Daniel Ciampi de Andrade, Rubens Gisbert Cury

**Affiliations:** 1 Universidade de São Paulo, Faculdade de Medicina, Departamento de Neurologia, São Paulo SP, Brazil.; 2 Clínica de Dor e Funcional, São Paulo SP, Brazil.; 3 Universidade Federal de São Paulo, São Paulo SP, Brazil.; 4 Hospital Geral de Fortaleza, Departamento de Neurologia, Centro de Distúrbios do Movimento, Fortaleza CE, Brazil.; 5 Universidade de Fortaleza, Faculdade de Medicina, Fortaleza CE, Brazil.; 6 West Virginia University, Camden Clark Medical Center, Parkersburg WV, United States.; 7 Hospital Universitário Cajuru, Curitiba PR, Brazil.; 8 Hospital Instituto de Neurologia de Curitiba (INC), Curitiba PR, Brazil.; 9 Hospital Sírio Libanês, Instituto de Ensino e Pesquisa, Brasília DF, Brazil.; 10 Universidade de Brasília, Hospital Universitário de Brasília, Serviço de Neurologia, Brasília DF, Brazil.; 11 Afya Centro Universitário Uninovafapi, Teresina PI, Brazil.; 12 Hospital Israelita Albert Einstein, São Paulo SP, Brazil.; 13 Centro Neuroon, Curitiba PR, Brazil.; 14 Universidade Federal Ciências da Saúde de Porto Alegre (UFCSPA), Porto Alegre RS, Brazil.; 15 Aalborg University, Faculty of Medicine, Department of Health Science and Technology, Center for Neuroplasticity and Pain (CNAP), Aalborg, Denmark.; 16 Universidade Federal do Rio de Janeiro, Rio de Janeiro RJ, Brazil.

**Keywords:** Deep Brain Stimulation, Dystonia, Brazil, Cohort Studies

## Abstract

**Background:**

Deep brain stimulation (DBS) is a treatment for dystonia, with most trials conducted in developed countries. Data from developing countries like Brazil are limited.

**Objective:**

To evaluate the landscape of DBS for dystonia in Brazil, assessing motor outcomes compared with the existing literature.

**Methods:**

A retrospective multicenter cohort study was conducted via medical record review. Demographics and motor outcomes were collected and analyzed using the Burke-Fahn-Marsden Dystonia Rating Scale (BFMDRS) from patients across four of Brazil's five macro-regions.

**Results:**

The cohort included 60 patients (44.3% female), with DBS primarily targeting the globus pallidus internus (73.3%) or subthalamic nucleus (18.3%). The BFMDRS motor scores decreased from 63.0 ± 26.2 (n = 24) at baseline to 36.7 ± 24.6 at 1-year post-DBS (n = 22, *p* = 0.0018) and 43.6 ± 35.0 at the last assessment (n = 13, *p* = 0.0327).

**Conclusion:**

The use of DBS yielded significant, sustained motor improvements, consistent with developed countries, highlighting its feasibility and effectiveness in Brazil within diverse healthcare settings.

## INTRODUCTION

Deep brain stimulation (DBS) is part of the treatment arsenal available for dystonia. Usually, DBS is indicated for people with dystonia (PwD) who are refractory to first-line treatments, such as botulinum toxin (BoNT) and oral medications. The primary targets are the globus pallidus internus (GPi) and the subthalamic nucleus (STN).

The pivotal trials and leading publications for DBS in dystonia were conducted in developed countries, particularly in Europe and the United States of America. However, data from developing countries, such as Brazil, remain scarce. This study aimed to establish a landscape of DBS treatment for dystonia in Brazil by evaluating the motor outcomes data available in our country and comparing them with the literature.

## METHODS

### Design

This was a retrospective multicenter cohort study conducted through medical records review. Our objective was to improve understanding of DBS surgery for dystonia in Brazil, including the associated motor symptoms outcomes. This study is part of the *Registro Brasileiro de Doenças Neurológicas* (REDONE.br) initiative of the Brazilian Neurology Academy (ABN, *Academia Brasileira de Neurologia* ).

### Patients and consent

This study protocol was approved by the coordinating center institutional Ethics Review Board - *Comissão de Ética para Análise de Projetos de Pesquisa* (CAPPesq), under the number #56808622.2.0000.0068.

Inclusion criteria comprised patients with any type of dystonia who had available medical records from January 1990 to April 2022, from various Brazilian public and private DBS outpatient clinics. Healthcare professionals who cared for these patients input data in the Research Electronic Data Capture (REDCap, Vanderbilt University) data management platform of the REDONE.br project.

### Data assessment

General and neurological clinical data were collected from medical records, including available motor scale assessments. Key variables included clinical and demographic variables, dystonia classification (age of onset, distribution, course, and variation), etiology, genetic markers, pharmacological treatments, use of BoNT, DBS targets, additional surgeries, and clinical outcomes assessed via standardized scales. The scales included the Burke-Fahn-Marsden Dystonia Rating (BFMDRS), Toronto Western Spasmodic Torticollis Rating (TWSTRS), and Unified Dystonia Rating Scale (UDRS). The variables were assessed at baseline, 1-year post-DBS, and at the last follow-up. Due to participation from various centers across Brazil, outcomes scales varied.

### Sample size and data analyses

This study employed a convenience sample approach. Data from single centers were collected and integrated for analysis and expressed as mean ± standard deviation (SD, min–max). All statistical calculations were performed using the IBM SPSS Statistics for Windows (IBM Corp.) software, version 28.0. Categorical data was analyzed through the Pearson Chi-squared test, and continuous data was assessed using the Kruskal-Wallis test. Normality was tested with the Kolmogorov-Smirnov and Shapiro-Wilk tests. Statistical significance was set at *p* < 0.05.

## RESULTS

### Demographics and clinical characteristics

Data from 60 patients were collected ( Table 1 ), with 44.3% (n = 27) being female. Patients were 42.1 ± 15.1-years-old (15.0–81.0) and had a mean dystonia duration of 20.8 ± 11.8 (4.5–52.4) years. Patients underwent DBS surgery an average of 15.9 ± 11.6 (0.8–46.0) years after symptoms onset. Our sample included patients from four out of five macro-regions of Brazil: Southeast (São Paulo state = 45, Rio de Janeiro state = 1), South (Paraná state = 10, Rio Grande do Sul state = 2), (Brasília = 1) and Northeast (Piauí state = 1, and Ceará state = 1).

**Table 1:** Sample characteristics

Demographic data		
Female		27 (44.3%)
Age (years)		42.1 ± 15.1 (15.0–81.0)
Dystonia duration (years)		20.8 ± 11.8 (4.5–52.4)
Time to DBS surgery (years)		15.9 ± 11.6 (0.8–46.0)
**Dystonia's characteristics**		
**Age at onset (n = 60)**	Infancy	5 (8.3%)
	Childhood	22 (36.7%)
	Adolescence	12 (20.0%)
	Early adulthood	12 (20.0%)
	Late adulthood	9 (15.0%)
**Distribution (n = 60)**	Focal	2 (3.3%)
	Generalized with lower limb	33 (55.0%)
	Generalized w/o lower limb	17 (28.3%)
	Hemidystonia	1 (1.7%)
	Multifocal	1 (1.7%)
	Segmental	6 (10.0%)
**Disease course (n = 60)**	Static	15 (25.0%)
	Progressive	45 (75.0%)
**Variability (n = 60)**	Action specific	1 (1.7%)
	Diurnal	1 (1.7%)
	Persistent	58 (96.7%)
**Associated feature (n = 57)**	Combined	8 (14.0%)
	Isolated	49 (86.0%)
**Other movement disorders**	Myoclonus	1 (25.0%)
	Parksonism	3 (75.0%)
**Etiology (n = 59)**	Acquired	12 (20.3%)
	Hereditary	21 (35.6%)
	Idiopathic	26 (44.1%)
**Genetics (n = 14)**	DYT- *TOR1A*	1 (7.14%)
	DYT- *SGCE*	1 (7.14%)
	DYT- *PRKRA*	3 (21.42%)
	DYT- *THAP1*	8 (57.14%)
	DYT- *KMT2B*	1 (7.14%)
**DBS targets (n = 60)**	GPi	44 (73.3%)
	GPi + STN	2 (3.3%)
	Gpi + VoA/VoP	1 (1.7%)
	Gpi + VoP	1 (1.7%)
	STN	11 (18.3%)
	VoP	1 (1.7%)

Abbreviations: BoNT, botulinum toxin; DBS, deep brain stimulation; GPi, *Globus pallidus internus* ; STN, subthalamic nucleus; VoA, ventral oral anterior nucleus; VoP, ventral oral posterior nucleus.

Data were collected from medical records across various healthcare settings, including private practices (19.7%), health insurance plans (3.3%), and public health services (77.0%).

Age at onset was classified as infancy (8.3%), childhood (36.7%), adolescence (20.0%), and early (20.0%) or late adulthood (15.0%). It was verified that 55.0% of patients' dystonia distribution was generalized with lower limb involvement, 28.3% without lower limb involvement, 10.0% segmental, 3.3% focal, 1.7% hemidystonia, and 1.7% multifocal dystonia. The disease course was progressive in 75.0% of the sample, and static in the other 25.0%. Isolated dystonia was noted in 86.0% (n = 49) of patients, while 14.0% had combined phenotypes, including myoclonus (25.0%) or parkinsonism (75.0%).

### Etiology and genetics

Etiology (n = 59) was classified as idiopathic (44.1%), hereditary (35.6%), or acquired (20.3%). Central nervous system evaluation (n = 55) showed no abnormalities in 87.3%, structural lesions in 9.1%, and neurodegeneration in 3.6%. Genetic testing (n = 14) identified DYT- *THAP1* (57.14%), DYT- *PRKRA* (21.42%), DYT- *TOR1A* (7.14%), DYT- *SGCE* (7.14%), and DYT- *KMT2B* (7.14%), as shown in Table 1 .

### Treatment modalities

**Figure 1:**
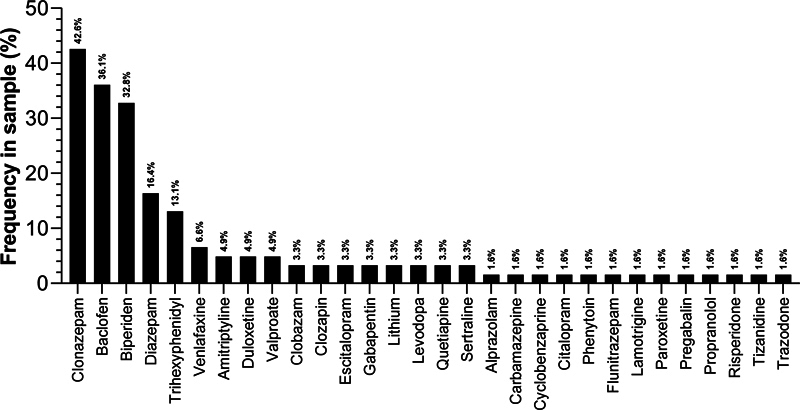
Types of medications used by patients.

Pharmacological management (n = 60) was diverse ( Figure 1 ), with clonazepam (42.6%), baclofen (36.1%), biperiden (32.8%), diazepam (16.4%), and trihexyphenidyl (13.1%) being the most prescribed. We administered BoNT in 98.3% (n = 58)of cases, of which 70.2% reported improvement ( *p* < 0.001).

### Surgical interventions

**Figure 2:**
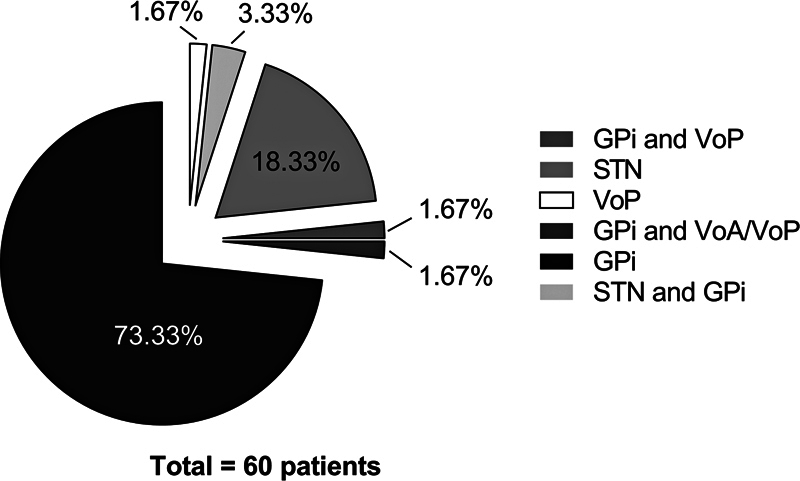
Abbreviations: GPi, *globus pallidus internus* ; STN, subthalamic nucleus; VoA, ventral oral anterior nucleus; VoP, ventral oral posterior nucleus. DBS targets.

The DBS surgery was performed on all patients, with the GPi as the primary target ( Figure 2 ) in 73.3% (n = 44), followed by the STN in 18.3% (n = 11) and the ventral oral posterior nucleus (VoP) in 1.7% (n = 1). Combined targets (each with n = 1, 1,7%) included GPi with STN, GPi with ventral oral anterior/posterior nuclei (VoA/VoP), and GPi with VoP.

Additional surgeries (n = 12) included pallidotomy (33.3%), electrode repositioning (25.0%), subthalamotomy (16.7%), baclofen pump implantation (8.3%), rhizotomy (8.3%), and battery replacement (8.3%). The last assessment by all 60 patients was 4.7 ± 3.3 (0.1–15.2) years after DBS surgery.

### Clinical outcomes

**Figure 3:**
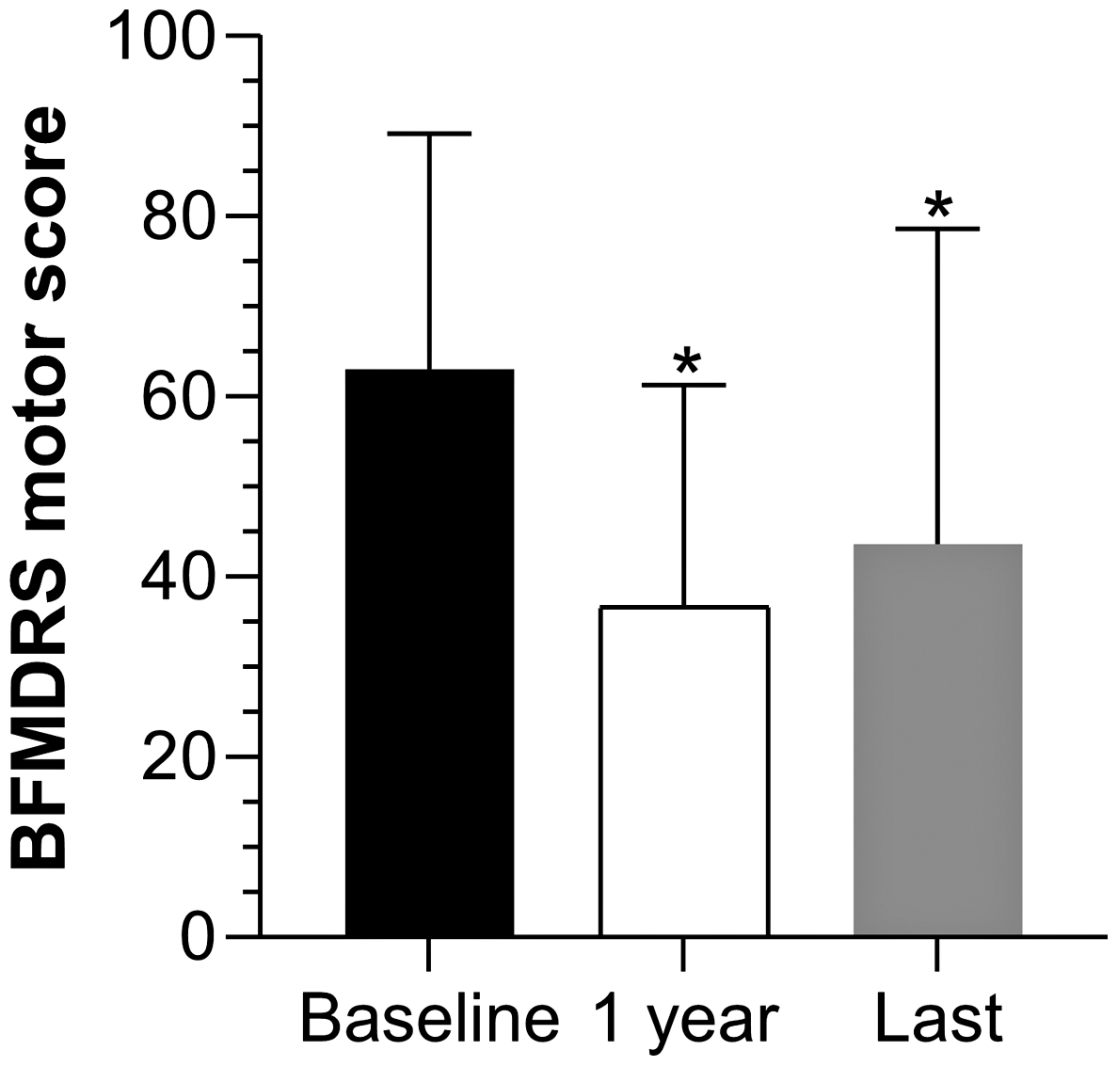
Abbreviations: BFMDRS, Burke-Fahn-Marsden dystonia rating scale; DBS, deep brain stimulation. Motor score outcomes. The figure shows the BFMDRS motor subscore at baseline, 1-year post-DBS, and the last follow-up.

Baseline BFMDRS motor scores (n = 24) averaged 63.0 ± 26.2 (21.0–112.0), with disability scores of 16.8 ± 8.5 (7.0–28.0), as reported in Table 2 and Figure 3 . At 1-year post-DBS (n = 22), motor scores decreased significantly to 36.7 ± 24.6 (1.0–112.0, *p* = 0.0018), and disability scores decreased to 10.2 ± 4.8 (4.0–21.0, *p* = 0.0924). At the last assessment (n = 13), motor scores were 43.6 ± 35.0 (6.0–112.0, *p* = 0.0327) when compared with baseline, and disability scores were 10.89 ± 7.6 (2.0–19.0; *p* = 0.0647, vs. baseline). No significant differences were observed between the 1-year and the last assessments ( *p* > 0.05). The proportion of patients with 50% improvement for BFMDRS motor scores were 35% after 1 year and 44% regarding the patients' last visit.

**Table 2:** Motor scores outcomes

	Baseline (n = 31)		1-year post-DBS (n = 26)			Last assessment (n = 18)		
Scale	n	Score	n	Score	*p* -value	n	Score	*p* -value
BFMDRS Motor	24	63.0 ± 26.2 (21.0–112.0)	22	36.7 ± 24.6 (1.0–112.0)*	0.0018	13 ^#^	43.6 ± 35.0 (6.0–112.0)*	0.0327
BFMDRS disability	24	16.8 ± 8.5 (7.0–28.0)	22	10.2 ± 4.8 (4.0–21.0)	0.0924	13 ^#^	10.89 ± 7.6 (2.0–19.0)	0.0647
UDRS	5	26.2 ± 7.5 (20.0–37.0)	3	15.7 ± 13.4 (6.0–31.0)	0.2091	4 ^!^	18.5 ± 10.9 (6.0–31.0)	0.3253
TWSTRS	2	22.0 ± 4.2 (19.0–25.0)	1	5.0 ± 0.0 (5.0–5.0)	0.1138	1 ^@^	6.0 ± 0.0 (6.0–6.0.0)	0.3428

Abbreviations: BFMDRS, Burke-Fahn-Marsden dystonia rating scale; DBS, deep brain stimulation; TWSTRS, Toronto Western spasmodic torticollis rating scale; UDRS, unified dystonia rating scale. Notes: * *p* < 0.05 vs. baseline using the Kruskal-Wallis test. ^#^ Group of patients: 9 = generalized inherited/idiopathic dystonia, 3 = generalized acquired dystonia, 1 = segmental idiopathic dystonia. ^!^ Group: 3 = generalized inherited/idiopathic dystonia, 1 = generalized acquired dystonia. ^@^ 1 patient with idiopathic focal dystonia.

The UDRS scores (n = 5 at baseline) dropped from 26.2 ± 7.5 (20.0–37.0) to 15.7 ± 13.4 (6.0–31.0) at 1-year (n = 3; *p* = 0.2091), and 18.5 ± 10.9 (6.0–31.0) at the last assessment (n = 4; *p* = 0.3253 *vs.* baseline). The TWSTRS scores (n = 2 at baseline) fell from 22.0 ± 4.2 (19.0–25.0), to 5.0 ± 0.0 at 1-year (n = 1; *p* = 0.1138), and 6.0 ± 0.0 at the last assessment (n = 1; *p* = 0.3428 vs. baseline).

Patients that had motor scores included in their last assessment in this cohort were evaluated an average of 2.0 ± 0.8 (1.3–4.9) years after DBS surgery.

## DISCUSSION

We included data from multiple states in our country with contrasting socioeconomic features. Our findings demonstrate significant motor improvements following DBS, with BFMDRS motor scores decreasing by 41.7% at 1-year and sustaining a decrease of 30.8% over an average 2-years of follow-up, consistent with reported outcomes in developed countries and other developing nations. The predominance of GPi as the primary DBS target (73.3%), alongside a notable use of STN-based stimulation (18.3%), reflects adherence to global standards, while alternative targets are explored in complex cases.

Previous studies on DBS for dystonia in Brazil included a prospective 1-year follow-up of 11 patients, also featured in the current study, in which both motor and non-motor symptoms were evaluated. There was an improvement of 47.9% in motor and 47.5% in non-motor symptoms. 1 The same institution evaluated motor performance in 5 dystonic patients, incorporated in this study, with stimulation of the STN region both before surgery and after it, at 7 months. 2

In 2018, Cury et al. 3 reviewed DBS outcomes for various dystonia types, primarily from developed countries. For idiopathic or inherited isolated segmental/generalized dystonia, BFMDRS motor score improvements ranged from 42 to 80%, with disability score improvements ranging from 23 to 70%. 3 A separate meta-analysis, with a mean follow-up of 32.5 months (range: 6–72 months), reported a mean average BFMDRS motor score reduction of 23.8 points at 6 months and 26.6 at the last follow-up, compared to baseline in isolated dystonia. 4 Cervical dystonia showed mean motor improvements of 47.6 to 70% in Cury's review. However, DBS motor outcomes combined dystonia (excluding myoclonus) or subtypes associated with other neurological or systemic conditions are generally less robust than in isolated cases, typically achieving less than 25% motor improvement, with tardive dystonia as a notable exception.

Motor score improvements in this cohort were slightly lower than those observed in idiopathic/inherited dystonias in developed countries. This may be due to several factors, including the greater heterogeneity in our sample (44.1% idiopathic, 35.6% hereditary, 20.3% acquired; Table 1 ), limited access to advanced surgical procedures, delayed surgical indication (mean: 15.9 years postonset), and longer disease duration (20.8 ± 11.8 years).

There is very little DBS data from developing countries in the literature. A Moroccan group described five patients with motor improvement measures through BFMDRS, ranging from 40 to 95%. 5 Another group from Malaysia examined a set of three patients with dystonia, of which two improved 96 and 68% in 2 years post-DBS, and the last one showed only 8.45% of improvement. 6

A multicenter study analyzed 67 dystonic patients with GPi-DBS from India, Kuwait, Egypt, and Turkey. 7 From that total, 30 patients had generalized dystonia (DYT- *TOR1A* : 20 and DYT- *TAF1* : 10), 27 had cervical dystonia (DYT-5: 17; and DYT- *THAP1* : 10), 5 had poststroke hemidystonia, and 5 had Parkinson's disease Pisa syndrome. Their mean BFMDRS improvement was 56 ± 1.0 at 6 to 12 months after DBS surgery, while patients had an average BFMDRS improvement of 26 ± 1.0 between 5 to 7 years postoperatively.

Rios et al. 8 used data from the Medtronic's Product Surveillance Registry from Latin America DBS centers (Colombia = 3, Argentina = 1, Brazil = 1, and Mexico = 1) between July 29, 2009, and July 31, 2021. 8 Dystonia was the second most frequent (18.62%) indication for DBS in this Latin America-focused study, while in Europe and the USA, it is the third most common after Parkinson's disease and essential tremor.

### Limitations

The main limitations of this study are its retrospective nature, the small sample size, heterogeneous sample and lack of long-term uniform follow-up and the fact that the available data were from medical records across different institutions, some of which apply distinct scales for motor outcomes. Though our initial aim was to do a subgroup analysis of the different types of dystonia, the small sample size (n = 60), with even smaller available motor outcome data (e.g., BFMDRS scores for n = 24 at baseline, n = 13 at last follow-up) restricted our ability to conduct meaningful comparisons without compromising statistical robustness. A formal subgroup analysis was not performed to avoid underpowering the statistical comparisons and risking unreliable conclusions.

Furthermore, although we managed to collect data from 60 patients, we acknowledge that this represents only a small portion from the population that have been submitted to DBS treatment for dystonia in Brazil. We hope to develop and approve a larger Database and Registry Study Group focusing on this condition in order to evaluate the different subtypes' response to DBS in Brazil.

In conclusion, this multicenter study provides the first comprehensive landscape of DBS for dystonia in Brazil, encompassing a diverse cohort of 60 patients across four of the country's five macro-regions. Our findings demonstrate significant motor improvements following DBS, with BFMDRS motor scores decreasing by 41.7% at 1-year ( *p* = 0.0018) and maintaining an average reduction of 30.8% after an overall follow-up of 2 years ( *p* = 0.0327 vs. baseline), aligning with outcomes reported in developed countries and other developing nations.

The slightly lower improvement than those of idiopathic/inherited generalized/segmental dystonias results from developed regions may be due to multiple reasons including our more heterogenous sample, limited access to advanced surgical procedures, delayed surgical indication, and longer disease duration. The predominance of GPi as the primary DBS target (73.3%), alongside a notable use of STN-based stimulation (18.3%), reflects adherence to global standards. Despite the retrospective nature of this study and the lack of standardized scales across centers, our results highlight the feasibility and effectiveness of DBS for dystonia in Brazil, even amidst socioeconomic diversity.
